# A high performance parallelizable MRI physics simulator with graphic processing unit technology

**DOI:** 10.1186/1532-429X-15-S1-E45

**Published:** 2013-01-30

**Authors:** Christos G Xanthis, Ioannis E Venetis, Anthony H Aletras

**Affiliations:** 1 Department of Computer Science and Biomedical Informatics, University of Central Greece, Lamia, Greece; 2 Department of Computer Engineering and Informatics, University of Patras, Patra, Greece

## Background

The acquisition of high quality Magnetic Resonance (MR) images requires exploring a parameter space for image quality improvement. This iterative process can benefit from simulations of MRI pulse sequences and imaging protocols.

Current MRI physics simulators are confined to few pulse sequences and compromises are made due to the high computational power needed. A step-by-step comprehensive Bloch equation simulation from signal acquisition to image formation, without the aforementioned compromises, may help in evaluating cardiovascular MRI protocols and pulse sequences.

## Methods

In this study, a comprehensive computer simulation platform of MR physics was designed and developed based on work by Nazarova et al (2004). The simulation platform makes no assumptions of the underlying pulse sequence and was developed in MATLAB. The computationally demanding core was executed in parallel within the graphic processing unit (GPU) environment by employing CUDA technology.

The simulation platform allowed the development of custom MRI pulse sequences and their application on computer models, including a detailed 3D model of the anatomy of the human heart and torso. The simulator accounted for the main static field and also allowed for the introduction of a time varying inhomogeneity field map. The time evolution of the magnetization vector and its components were displayed in 3D.

The efficacy of GPU implementation was evaluated against other computer configurations. Execution times were recorded for the application of the simulator on three different computer configurations a) one single CPU computer (Intel Pentium D, 3.40GHz) b) a 23 core computer cluster and c) one single CPU computer with one GPU Tesla c2070 of 448 GPU cores. These simulations were based on a Gradient Echo pulse sequence applied on a cubic object of 21600 voxels (k-space 256x256).

Finally, the GPU-based simulation was also applied on a detailed 3D model of the anatomy of the human heart. However, due to RAM memory size limitations on the PC side (4 GB), it was applied only to the right atrium tissue and its blood cavity.

## Results

Speedup of almost three orders of magnitude (x908) was recorded when compared to CPU-based and more than two orders of magnitude (x240) when compared to cluster-based systems.

**Figure 1:**
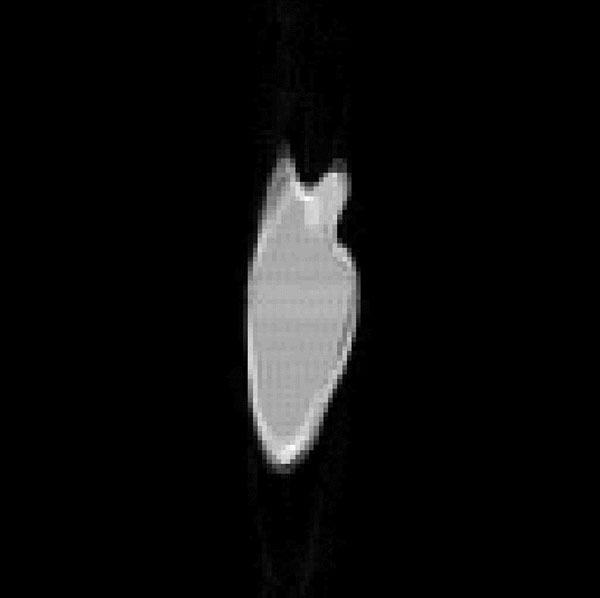
Simulated short-axis tomographic image of the right atrium and its blood cavity.

A simulated short-axis tomographic image of the right atrium and its blood cavity is shown in Figure 1.

## Conclusions

The MRI simulator proposed in this study allows its application in large-scale analysis without model simplifications by employing the GPU technology. The almost three orders of magnitude speedup obtained in MRI simulations with a single video card of 448 GPU cores is promising. The future reduction in cost of GPU based technology suggests that such an MRI simulator has the potential to become an indispensable tool for medical imaging centers and should change the manner by which MRI protocols are optimized.

## Funding

Funding provided by the European Research Council via a four year Marie Curie Actions grant (FP7 - PEOPLE - 2009 - IRG call) and by the ‘Alexander S. Onassis public benefit foundation'.

